# Health-Promoting Behaviors, Physical Self-Efficacy, Exercise Adherence, and Sports Commitment Among Older Adults Who Participate in Sports Activities

**DOI:** 10.3390/healthcare12212135

**Published:** 2024-10-26

**Authors:** Seung-Hwan Woo, Jae-Pil Seo, Hyun-Ryun Kim, Wi-Young So, Young-Kyun Sim

**Affiliations:** 1 College of Convergence in Culture, U1 University, Yeongdong-gun 29131, Republic of Korea; woosh0916@u1.ac.kr; 2 Sports & Leisure Studies, Gimcheon University, Gimcheon-si 39528, Republic of Korea; 0025@gimcheon.ac.kr; 3 Department of Physical Education, Woosuk University, Wanju-gun 55338, Republic of Korea; khr870615@woosuk.ac.kr; 4 Department of Sports Medicine, College of Humanities, Korea National University of Transportation, Chungju-si 27469, Republic of Korea

**Keywords:** exercise psychology, health promotion, healthy life, older adults, super-aged society

## Abstract

Objectives: The Republic of Korea is progressively becoming a super-aged society, emphasizing the need for regular physical activity among older adults because it has physical, psychological, and social benefits. Recently, increasing depression and suicide rates have been reported among older adults living alone. However, research that considers older adults’ living situations is limited. Therefore, this study aimed to analyze the relationship between health-promoting behaviors (HPB), physical self-efficacy (PSE), exercise adherence, and sports commitment among older adults aged ≥ 65 years who participated in sports activities and investigate the influence of their living situation. Methods: The participants were 452 individuals aged ≥ 65 who lived in the metropolitan areas of Chungcheong-do, Jeolla-do, and Gwangju Provinces, Republic of Korea, and who regularly participated in sports activities (men = 283, women = 169). This study was conducted from January to May 2024. Data were collected using a structured and validated questionnaire, and the collected data were analyzed using descriptive statistics, correlation analysis, structural equation modeling, and multi-group analysis. Results: Structural equation modeling showed that the research model was appropriate, and all five paths showed statistical significance. The identity of the model was verified in the multi-group analysis, but path coefficients differed between older adults living alone and those living with family members. HPB significantly affected PSE, exercise adherence, and sports commitment among both groups of older adults. However, the impact of PSE on exercise adherence and sports commitment was not statistically significant among older adults living alone. Conclusions: The results highlight the need to consider older adults’ living situations when establishing regular physical activity. Efforts should also be made to promote regular exercise participation among older adults living alone.

## 1. Introduction

The Republic of Korea is on the verge of becoming a super-aged society, necessitating action to address several issues, such as the health problems of older adults, increased medical costs, and pension systems for older individuals [1,2]. Healthy living through regular physical activity is drawing attention as a solution to these problems, as physical activity in old age improves physical, psychological, and social health [3]. Physically, it helps prevent chronic diseases, such as heart disease, stroke, high blood pressure, diabetes, and dementia. Psychologically, it helps control depression and anxiety experienced in old age [4], and socially, it helps tackle social isolation. Older adults with reduced physical function become socially isolated when they lose their spouse, face economic difficulties, or lack communication with their family [5,6]. In addition, physical inactivity contributes to social disconnection and creates a vicious cycle of accelerated aging due to poor physical fitness [7].

However, to solve the health-related problems of older adults, their living situation should be considered, as health-related problems differ significantly based on whether one lives alone or with family members [8,9]. Older adults living with their spouse or family members are less likely to feel socially isolated, as they have social relationships and social support. In contrast, those living alone will likely experience social isolation owing to a lack of social relationships and support [10,11]. Complex factors, such as the living situation and the presence of illness, make it challenging to address older adults’ health-related problems [12]. The illnesses mainly experienced by older adults are chronic or degenerative, which require prevention rather than continuous management [2].

Health-promoting behaviors (HPBs), which help maintain health, represent one approach to disease prevention [13,14]. Some examples of HPBs are engaging in regular exercise, quitting smoking and drinking, consuming a balanced diet, and getting vaccinated. However, the performance of HPBs is influenced by individual characteristics, such as perceived health status, physical ability, and past experiences, which could be positive or negative [15]. Nonetheless, the performance of HPBs provides health benefits and improves perceived self-efficacy, as a person’s confidence in their physical ability increases with expectations of positive outcomes (i.e., a healthy life). Therefore, participating in physical activity (an HPB) and experiencing changes in physical status can improve an individual’s confidence in their physical ability, referred to as physical self-efficacy (PSE).

PSE is derived from the self-efficacy theory [16,17] and is defined as the expectation and confidence level to complete a given task successfully. The expectation and belief that one can succeed leads to a positive outcome [18]. Furthermore, a high level of self-efficacy acts as a driving force for sustained behavior due to the focus and anticipation of the task. PSE comprises two sub-factors: perceived physical ability and physical self-presentation confidence [19,20]. Perceived physical ability refers to an individual’s perceptions about their ability to perform physical activities or tasks, and physical self-presentation confidence is one’s confidence in performing physical activities that are to be evaluated by others [21,22]. Given that HPBs include performing regular physical activity, they can be considered to be closely related to PSE. In fact, many studies have demonstrated a relationship between HPBs and PSE [20,23,24,25]. Considering that HPBs encompass those behaviors that facilitate a healthy life, they are likely to induce exercise adherence.

Exercise adherence refers to the level of one’s attachment and obsession with a particular physical activity. High levels of exercise adherence denote a state in which one does not intend to give up exercising regularly [26,27], and individuals with high levels of exercise adherence consider exercising a part of their lives. Older adults who recognize the importance of a healthy life may consider the benefits of physical activity and engage in it, thus fostering exercise adherence. In fact, many studies have shown a relationship between HPBs and exercise adherence [20,28] and report that self-efficacy for physical activity determines the frequency of exercise participation among older adults [29]. This indicates that older adults’ PSE influences their exercise adherence and commitment to regular physical activity [30]. However, participation in physical activity among older adults in the Republic of Korea is declining [31], and researchers are calling for urgent measures to maximize interest in physical activity. In this regard, various physical activity programs should be formulated and implemented to stimulate behavioral change by triggering persistence in exercise [32,33].

Furthermore, participating in regular physical activity leads to sports immersion/commitment, a state of cognitive and behavioral engagement [34]. Individuals become engrossed in their physical activities and invest high levels of effort and time. Cognitive commitment results from feelings of fun and interest in physical activity, whereas behavioral commitment emerges from passion for physical activities [35]. According to previous studies, the performance of HPBs promotes sports engagement, and PSE is positively related to sports commitment [36,37]. These findings indicate that HPBs and PSE influence sports commitment.

**Figure 1:**
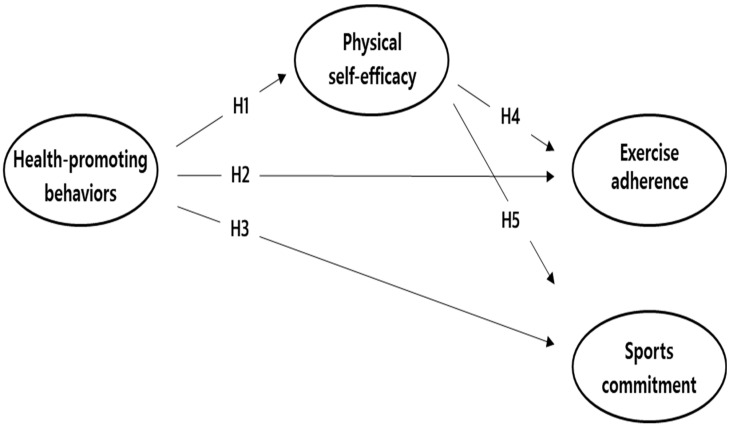
Hypothesized model.

Numerous studies have examined HPBs, but such research remains limited among older adults in Asia. Scholars assert that older individuals who live alone exhibit high rates of depression and suicide [3,5], suggesting that HPBs and exercise adherence may differ depending on whether an older individual lives alone or with family members. However, few studies have considered the living situation of older adults. Empirical research is needed to examine the relationship between HPBs, PSE, exercise adherence, and sports commitment and investigate this relationship based on older adults’ living situation. This study employed a multi-group analysis and examined the relationship between HPBs, PSE, exercise adherence, and sports commitment among older adults who participate in sports activities based on whether they live alone or with family members. Figure 1 presents the hypothesized model.

**H1.**  *Health-promoting behaviors will influence physical self-efficacy*.

**H2.**  *Health-promoting behaviors will influence exercise adherence*.

**H3.**  *Health-promoting behaviors will influence sports commitment*.

**H4**  . *Physical self-efficacy will influence exercise adherence*.

**H5.**  *Physical self-efficacy will influence sports commitment*.

## 2. Materials and Methods

### 2.1. Participants

This study was conducted from January to June 2024. It targeted individuals aged ≥ 65 who lived in the metropolitan areas of Chungcheong-do, Jeolla-do, and Gwangju Provinces, Republic of Korea, and who participated in sports activities. To collect the data, the researcher explained the purpose of this study over the phone to the officials of the sports clubs located in the target area and sought their prior consent to conduct the study. Three researchers personally visited the institutions that agreed. Prior to the survey, given that the study involved older adults, the research team explained the purpose and intent of the study and obtained consent from each individual. Paper questionnaires were then distributed to the 500 people who volunteered to participate and were completed through a self-administered method that required them to fill in the survey responses themselves and were collected by the researcher at the end of the survey. The questionnaires took up to 10 min to complete.

In addition, to comply with the ethical requirements of the study, participants were informed that all survey data would be anonymized and would not be used for any purpose other than academic research and that their information would be kept strictly confidential. This study excluded 48 responses owing to inaccurate answers and used the responses of 452 participants (men = 283, women = 169).

### 2.2. Measures

The research instrument was a structured questionnaire, and all variables and questions were based on previous research that aligned with the objectives of this study. HPBs were measured using a scale developed by Walker et al. [37] that Sim and Seo [29] adapted to the Korean culture and context. It comprises eleven items across three factors: health management (three items, e.g., “I eat proper nutrition every day” and “I take regular breaks every day”), internal and external relationships (four items, e.g., “I participate in various clubs, societies, etc.” or “I always like to be in a group rather than alone”), and health promotion activities (four items, e.g., “I feel healthier when I’m sweating and out of breath” or “I tend to exercise regularly”). PSE was measured using a scale developed by Ryckman et al. [38], and Kim and Oh [39] validated to fit the Korean culture and context. It consists of nine items across two factors: perceived physical ability (four items, e.g., “I still consider myself physically fit” and “My focus is on physical activity”) and physical self-presentation confidence (five items, e.g., “I don’t worry about what other people think about my physique” and “I have a big voice and I have a good voice”). Exercise adherence was measured using a test developed by Courneya and McCauley [40] in Poff’s [41] study that Yang [42] adapted to the Korean culture and context. It comprises five single-factor questions (e.g., “I want to keep doing this exercise no matter what” and “I want to do this exercise when I have time”). Sports commitment was measured using a scale developed by Scanlan et al. [34] that was validated by Jung [43] to fit the Korean culture and context. It consists of eleven items across two factors: cognitive commitment (seven items, e.g., “I get a lot of happiness from this exercise” and “I get excited when I think about this exercise”) and behavioral commitment (four items, e.g., “I try to stay informed about the exercise I am participating in” and “I sometimes imagine myself doing this exercise in a cool way”).

Before using the questionnaire, its content validity was reviewed by a group of experts comprising one professor specializing in sports activities, one professor with research experience concerning older adults, and one doctor experienced in developing measurement tools. They repeatedly reviewed the items, checked their content validity, and modified and supplemented them to make them suitable for individuals aged ≥ 65. Specifically, the questionnaire consisted of four items on sociodemographic characteristics (sex, age, weekly frequency of participation in sports activities, and living situation), eleven items on the three factors of HPBs, nine items on the two factors of PSE, five items on exercise adherence, and eleven items on the two factors of sports commitment, making a total of 40 items across eight factors. All items were rated on a 5-point Likert scale, and the response options were “not at all,” “not really,” “average,” “yes,” and “very much.”

### 2.3. Data Analysis

The collected data were analyzed using SPSS and AMOS for Windows (version 23.0; IBM Corp., Armonk, NY, USA). First, descriptive statistics were calculated to determine the normality of the collected data. The mean and standard deviation (SD) were calculated, and normality was examined using skewness and kurtosis values. Following Kline’s criteria [44], the data were considered normally distributed if the skewness value was ± 3.00 or less and the kurtosis value was ±8.00 or less. Second, we conducted a confirmatory factor analysis (CFA) using the maximum likelihood estimation method and calculated Cronbach’s ɑ coefficients to determine the validity and reliability of the measurement tool. Following Kline’s criteria [44], the model fit criteria were judged based on the χ^2^/df (<0.300), Tucker–Lewis index (TLI) (>0.900), comparative fit index (CFI) (>0.900), root mean square error of approximation (RMSEA) (<0.080), and standardized root mean square residual (SRMR) (<0.080). However, as model fit criteria are not absolute values, even if an approximate value is derived, it can be considered to indicate an “acceptable fit.” Regarding reliability, Cronbach’s ɑ coefficient ≥ 0.60 indicated internal consistency [45]. Furthermore, the average variance extracted (AVE) and composite reliability (CR) were used to measure convergent validity. AVE values ≥ 0.500 and CR values ≥ 0.700 denoted a good fit [46]. Third, a correlation analysis was conducted to check for multicollinearity between the measurement variables. In the case of Pearson correlation coefficients, the closer the coefficient value is to 1.000, the higher the correlation. Fourth, the fit of the measurement model was verified based on the same criteria as those of the CFA model [44]. Subsequently, the measurement model fit was sequentially analyzed based on the base model (unconstrained), which freely estimates all paths; the measurement identity (measurement weights), which equally controls the factor structure between groups; and the structural identity model (structural weights), which equally controls the structural paths. This was performed to determine whether the relationships between the variables the research model intended to measure were verified under the same conditions. Model selection was judged using the relative model fit verification method and chi-squared test statistics. The CFI, TLI, and RMSEA values were used as relative model fit indices. If ΔCFI was less than 0.01, the model fit was judged to be the same [47]. In other words, if the CFI was less than 0.01 in the model with the same constraints as the previous model, the model with the equality constraints was adopted. Model selection was not performed based on the value of χ^2^ because the null hypothesis tends to be rejected when the sample size is large. Finally, structural equation modeling was conducted to determine the relationships between HPBs, PSE, exercise adherence, and sports commitment, and a multi-group analysis was conducted based on the participants’ living situations. The model fit was determined based on the χ^2^/df, TLI, CFI, RMSEA, and SRMR values commonly used by researchers [44]. Subsequently, unstandardized (B) and standardized coefficients (β) were calculated to verify the path coefficient. Statistical significance was set at *p* < 0.05.

## 3. Results

### 3.1. Sociodemographic Characteristics of the Participants

The participants had an average age of 69.94 years (SD = 5.75), and they participated in sports activities more than 3.29 times a week (SD = 1.44). Regarding their living situation, 204 participants lived alone, and 248 lived with a family member. Table 1 presents the sociodemographic characteristics of the participants.

### 3.2. Descriptive Statistics and Validity and Reliability of the Measurement Tool

The mean of the variables ranged from 3.334 to 4.216 points (Table 2). The SD ranged from 0.511 to 0.911 points. The skewness values ranged from −0.266 to −1.138 points, and kurtosis values ranged from 0.135 to 2.738 points, indicating normal distribution. Specifically, internal and external relationships had the highest mean at 4.216 points, while perceived physical ability had the lowest mean at 3.334 points. And, the results of confirmatory factor analysis and reliability analysis in Table 3.

### 3.3. Correlation Analysis

Significant correlations were found between all sub-factors (Table 4). In particular, health promotion activities and cognitive commitment showed the strongest positive association, with a correlation coefficient of 0.662 (*p* < 0.01), whereas physical self-presentation confidence and health management showed the weakest association, with a correlation coefficient of 0.096 (*p* < 0.05). Most importantly, because the correlation coefficients did not exceed 0.800, there were no concerns about multicollinearity [44].

### 3.4. Measurement Model Invariance

To verify the measurement model, we sequentially analyzed unconstrained, measurement weights, and structural weights models (Table 5). The fit of the measurement weights model with factor loading constraints (χ^2^ = 347.006, df = 106, TLI = 0.882, CFI = 0.905, RMSEA = 0.071) was better than that of the unconstrained model (χ^2^ = 332.222, df = 98, TLI = 0.876, CFI = 0.908, and RMSEA = 0.073). Subsequently, the model with the identity constraint on the path coefficient and the base model were compared to confirm significant differences among the path coefficients of the groups. The fit of the structural weights model was χ^2^ = 384.279, df = 123, TLI = 0.889, CFI = 0.897, and RMSEA = 0.069. Then, the CFI values of the measurement identity and structural identity models were compared. This was performed because, in the case of χ^2^, an increase in the number of samples affects the derivation of the results. Because ΔCFI was less than 0.01, the fit of the two models was interpreted under the same conditions [47]. Therefore, we adopted structural weights with identity constraints between path coefficients.

### 3.5. Structural Equation Modeling

**Figure 2:**
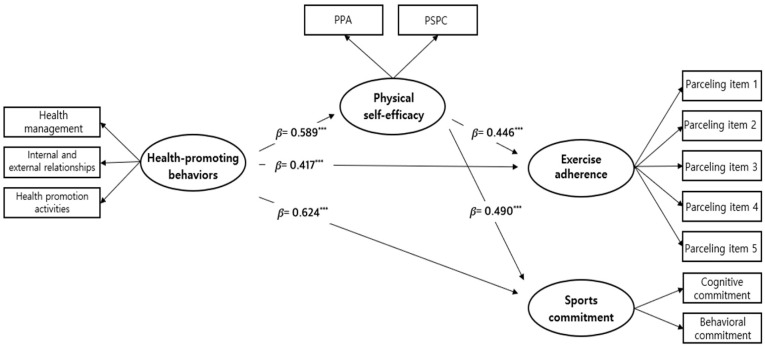
Standardized estimates of the research model (*** *p* < 0.001). PPA, Perceived Physical Ability; PSPC, Physical Self-Presentation Confidence

Following Kline’s criteria [44], the model fit was considered acceptable based on the following indices: χ^2^/df = 2.406 (χ^2^ = 202.084, df = 49), CFI = 0.893, TLI = 0.855, SRMR = 0.063, and RMSEA = 0.073 (95% confidence interval = 0.132, 0.105). Table 6 and Figure 2 present the results of verifying the hypotheses. HPBs had a positive effect on PSE (β = 0.589, *p* < 0.001), exercise adherence (β = 0.417, *p* < 0.001), and sports commitment (β = 0.624, *p* < 0.001). Furthermore, PSE positively influenced exercise adherence (β = 0.446, *p* < 0.001) and sports commitment (β = 0.490, *p* < 0.001).

### 3.6. Multi-Group Analysis

**Figure 3:**
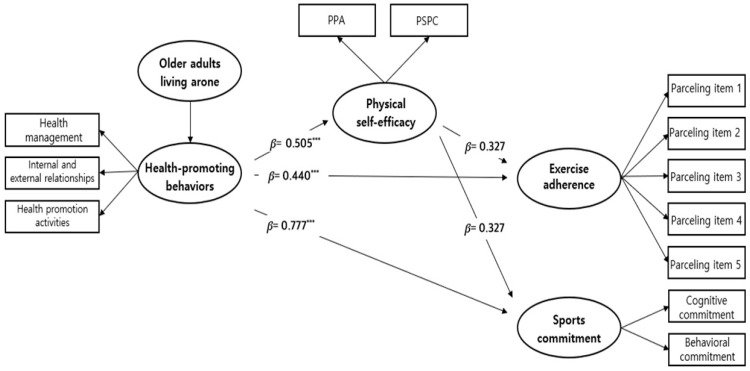

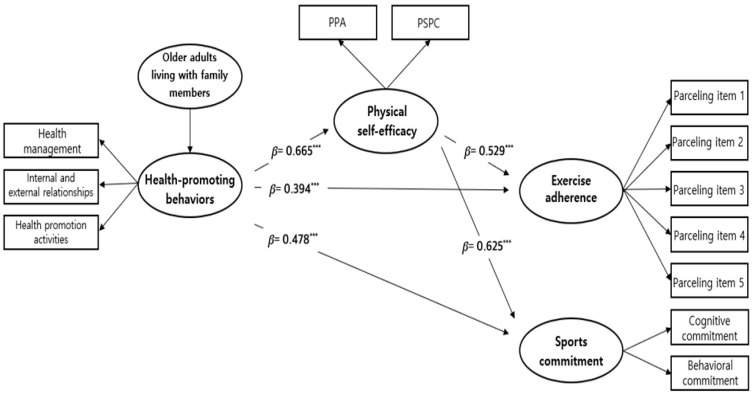
Standardized estimates of the research model in the multi-group analysis (*** *p* < 0.001) PPA, Perceived Physical Ability; PSPC, Physical Self-Presentation Confidence.

The path coefficient results of the structural identity model are presented in Table 7 and Figure 3. HPBs had a significant effect on PSE among participants who lived alone (β = 0.505, *p* < 0.001) and those who lived with family members (β = 0.665, *p* < 0.001). HPBs had a significant effect on exercise adherence among those who lived alone (β = 0.440, *p* < 0.001) and those who lived with family members (β = 0.394, *p* < 0.001). Similarly, HPBs significantly affected sports commitment among participants who lived alone (β = 0.777, *p* < 0.001) and those who lived with family members (β = 0.478, *p* < 0.001). However, PSE significantly affected exercise adherence (β = 0.529, *p* < 0.001) and sports commitment (β = 0.625, *p* < 0.001) only among participants who lived with family members.

## 4. Discussion

### 4.1. Interpretation of the Findings

Considering Korea is fast progressing toward a super-aged society, discussions on older adults’ participation in regular physical activity are ongoing. This is because regular physical activity helps control physical, psychological, and social decline. As part of these efforts, this study verified the relationship between HPBs, PSE, exercise adherence, and sports commitment among older adults who participate in sports activities and compared the relationships among those who live alone and those who live with family members.

The results showed that HPBs significantly affect PSE, exercise adherence, and sports commitment, consistent with previous findings conducted in Korea [32,48]. Those studies found that HPBs lead to exercise adherence and influence self-efficacy by facilitating positive recognition of oneself. The standardized regression coefficient, which indicates the influence of the variables, was also significant. These results imply that older adults who recognize the importance of HPBs enhance their PSE, which is an important factor in promoting exercise adherence and sports commitment [48]. Nevertheless, the development of HPB needs to be considered to promote the continued participation and engagement of older adults in exercise. HPB is influenced by the older adults’ past exercise experiences, their perceived self-efficacy level, and the accrued health benefits. Therefore, rather than simply encouraging people to engage in physical activity, creating conditions that make it more convenient and less financially burdensome is important. For example, a physical activity environment should be created close to the place of residence. Furthermore, providing financial support may help reduce the financial burden of participating in physical activity.

In addition, the present study results showed that PSE improves exercise adherence and sports commitment among older adults who participate in sports activities, as reported previously [35,36]. The findings indicate that the higher the PSE during sports activities, the more likely one is to continue exercising and become engrossed in it. As PSE is similar to the concept of self-efficacy proposed by Bandura [16], exercise adherence may have stemmed from the belief that one can successfully perform a physical activity and the positive results that emanate from performing the physical activity. Furthermore, engaging in and systematically performing an appropriate physical task increases the possibility of being immersed in the activity. This approach can be used to promote sports activities among older adults.

However, the multigroup analyses of older adults living alone or with family showed statistically significant results. In the group of older adults living with family, the pathways between HPB and PSE and the relationship between exercise persistence and exercise engagement were significant. However, the relationship between PSE and exercise persistence and exercise engagement was not statistically significant in the group of older adults living alone. This suggests that the level of persistence and engagement in exercise is different in the group of older adults living alone compared to the group of older adults living with family. These results support the findings of other studies conducted on older adults who do or do not live alone [3,12]. These studies shed light on the lives of older adults living alone because they experience difficulties overcoming social isolation due to the breakdown of social relationships and an environment that excludes social support. This is reflected in the results of this study. In other words, older adults who live alone are likely limited in their participation in continuous exercise due to social isolation and social disconnection. To compensate for these problems, ways to stimulate continued exercise participation based on social support and interest may be identified for older adults living alone.

Overall, the results of this study are salient because they clarify the role of HPBs among older Korean adults who participate in sports activities. They show that HPBs increase PSE, exercise adherence, and sports commitment. Above all, they emphasize the need to focus on the exercise adherence of older adults who live alone. Considering that older adults’ healthy lives are a prominent issue that needs attention, the present study results may be useful in formulating health promotion strategies.

### 4.2. Limitations and Directions for Future Research

This study has some limitations. Because of the characteristics of structural equation modeling, which requires simplicity of the research model, an analysis of the sub-factors could not be conducted. In essence, structural equation modeling verifies theoretical relationships or structures, but additional research is needed to determine causal relationships between variables. Therefore, multiple regression analysis of the sub-factors or expansion of the research model is needed. Furthermore, while this study emphasized the presence or absence of living alone, future research should conduct multi-group analyses considering factors such as gender and income level. In particular, as observed in previous studies [48,49], stratifying the population according to gender may reach different conclusions compared to the results of this study. Through such studies, in-depth information about HPBs, PSE, and exercise adherence can be gathered, which can help increase sports commitment among older adults.

## 5. Conclusions

Among older adults who participated in sports activities, HPBs predicted PSE and affected exercise adherence and sports commitment directly and indirectly. Exercise adherence and sports commitment differed according to whether the older adults lived alone or with family members. To increase HPBs, interest and support in physical activity must be increased, focusing on promoting exercise adherence among older adults who live alone. The present study results will likely increase interest in exercise and exercise adherence among older adults.

## Figures and Tables

**Table 1:** Sociodemographic characteristics of the participants (n = 452).

Characteristic	Category	Frequency	Percentage
Sex	Men	283	62.61
	Women	169	37.39
Age	65–69 years	227	50.22
	70–79 years	204	45.13
	≥80 years	21	4.65
Weekly frequency of participation in sports activities	1 day	41	9.07
	2–4 days	325	71.90
	≥5 days	86	19.03
Living situation	Living alone	204	45.13
	Living with family members	248	54.87
Total		452	100.00

**Table 2:** Descriptive statistics of the factors.

Factor	Mean	Standard Deviation	Skewness	Kurtosis
Health management (points)	3.741	0.664	−0.266	0.135
Internal and external relationships (points)	4.216	0.568	−0.464	0.676
Health promotion activities (points)	4.038	0.712	−1.138	2.738
Perceived physical ability (points)	3.334	0.615	−0.335	0.181
Physical self-presentation confidence (points)	3.344	0.862	0.407	−0.479
Exercise adherence (points)	3.350	0.511	0.718	2.735
Behavioral commitment (points)	3.355	0.911	−0.763	0.354
Cognitive commitment (points)	4.095	0.633	−0.711	2.199

**Table 3:** Results of confirmatory factor analysis and reliability analysis.

Latent Variable		Measurement Variable		B	β	S.E.	t	AVE	CR	Cronbach’s α	Model Fit
Health-promoting behaviors	Health management	→	Item 1	1.000	0.896	-	-	0.770	0.944	0.944	χ^2^ = 108.523, df = 24 TLI = 0.930 CFI = 0.954 SRMR = 0.062 RMSEA = 0.078
		→	Item 2	0.954	0.879	0.035	27.469 ***				
		→	Item 3	0.972	0.866	0.037	26.607 ***				
		→	Item 4	0.962	0.863	0.036	26.419 ***				
		→	Item 5	0.971	0.884	0.035	27.831 ***				
	Internal and external relationships	→	Item 6	1.000	0.889	-	-	0.767	0.943	0.942	
		→	Item 7	1.013	0.886	0.037	27.479 ***				
		→	Item 8	1.067	0.928	0.035	30.776 ***				
		→	Item 9	1.020	0.856	0.040	25.683 ***				
		→	Item 10	1.013	0.816	0.043	23.372 ***				
	Health promotion activities	→	Item 11	1.000	0.846	-	-	0.774	0.945	0.944	
		→	Item 12	1.040	0.901	0.040	25.758 ***				
		→	Item 13	1.068	0.930	0.039	27.279 ***				
		→	Item 14	1.008	0.871	0.042	24.210 ***				
		→	Item 15	0.998	0.848	0.043	23.102 ***				
Physical self-efficacy	Perceived physical ability	→	Item 1	1.000	0.869	-	-	0.612	0.862	0.908	χ^2^ = 126.172, df = 13 TLI = 0.887 CFI = 0.930 SRMR = 0.049 RMSEA = 0.038
		→	Item 2	0.994	0.849	0.042	23.795 ***				
		→	Item 3	0.827	0.724	0.051	16.298 ***				
		→	Item 4	0.816	0.670	0.042	19.232 ***				
	Physical self-presentation confidence	→	Item 5	1.000	0.878	-	-	0.653	0.882	0.890	
		→	Item 6	0.957	0.892	0.047	20.411 ***				
		→	Item 7	0.833	0.681	0.049	16.935 ***				
		→	Item 8	0.746	0.763	0.049	15.301 ***				
Exercise adherence		→	Item 1	1.000	0.867	-	-	0.612	0.884	0.885	χ^2^ = 151, df = 5 TLI = 0.935 CFI = 0.968 SRMR = 0.076 RMSEA = 0.033
		→	Item 2	0.983	0.889	0.039	25.401 ***				
		→	Item 3	1.012	0.907	0.039	26.208 ***				
		→	Item 4	0.573	0.544	0.046	12.337 ***				
		→	Item 5	0.596	0.633	0.040	14.996 ***				
Sports commitment	Cognitive commitment	→	Item 1	1.000	0.917	-	-	0.796	0.959	0.953	χ^2^ = 209, df = 26 TLI = 0.923 CFI = 0.944 SRMR = 0.064 RMSEA = 0.051
		→	Item 2	0.977	0.909	0.030	32.184 ***				
		→	Item 3	0.957	0.902	0.030	31.505 ***				
		→	Item 4	0.957	0.869	0.034	28.545 ***				
		→	Item 5	0.931	0.878	0.032	29.291 ***				
	Behavioral commitment	→	Item 6	1.000	0.857	-	-	0.600	0.856	0.859	
		→	Item 7	1.012	0.838	0.052	19.560 ***				
		→	Item 8	0.931	0.697	0.059	15.810 ***				
		→	Item 9	0.918	0.691	0.059	15.634 ***				

S.E., standard error; AVE, average variance extracted; CR, composite reliability; TLI, Tucker–Lewis index; CFI, comparative fit index; SRMR, standardized root mean square residual; RMSEA, root mean square error of approximation *** *p* < 0.001; tested using confirmatory factor analysis and Cronbach’s α.

**Table 4:** Correlation coefficients of the sub-factors.

Sub-Factor	1	2	3	4	5	6	7	8
1. Health management	1.000							
2. Internal and external relationships	0.217 **	1.000						
3. Health promotion activities	0.353 **	0.529 **	1.000					
4. Perceived physical ability	0.176 **	0.174 **	0.316 **	1.000				
5. Physical self-presentation confidence	0.096 *	0.120 *	0.093 *	0.261 **	1.000			
6. Exercise adherence	0.185 **	0.208 **	0.392 **	0.313 **	0.195 **	1.000		
7. Behavioral commitment	0.288 **	0.156 **	0.409 **	0.467 **	0.110 *	0.440 **	1.000	
8. Cognitive commitment	0.198 **	0.527 **	0.662 **	0.366 **	0.110 *	0.382 **	0.405 **	1.000

* *p* < 0.05, ** *p* < 0.01; tested using Pearson correction analysis.

**Table 5:** Results of analyzing measurement model invariance.

Model	χ^2^	df	*p*	χ^2^/df	Tucker–Lewis Index	Comparative Fit Index	Root Mean Square Error of Approximation
Unconstrained	332.222	98	<0.001	3.390	0.876	0.908	0.073
Measurement weights	347.006	106	<0.001	3.274	0.882	0.905	0.071
Structural weights	384.279	123	<0.001	3.124	0.889	0.897	0.069

**Table 6:** Results of verifying the hypotheses.

Path			B	*β*	Standard Error	*t*	Hypothesis
Health-promoting behaviors Health-promoting behaviors Health-promoting behaviors Physical self-efficacy Physical self-efficacy	→	Physical self-efficacy	0.494	0.589	0.052	8.333 ***	Supported
	→	Exercise adherence	0.359	0.417	0.077	4.875 ***	Supported
	→	Sports commitment	0.489	0.624	0.074	6.851 ***	Supported
	→	Exercise adherence	0.458	0.446	0.102	4.412 ***	Supported
	→	Sports commitment	0.457	0.490	0.108	4.511 ***	Supported

*** *p* < 0.001; tested using structural equation modeling.

**Table 7:** Results of the multi-group analysis.

Path			Living Alone				Living with Family Members			
			B	β	Standard Error	t	B	β	Standard Error	t
Health-promoting behaviors	→	Physical self-efficacy	0.559	0.505	0.106	5.273 ***	0.469	0.665	0.070	6.701 ***
Health-promoting behaviors	→	Exercise adherence	0.371	0.440	0.096	3.866 ***	0.348	0.394	0.102	3.405 ***
Health-promoting behaviors	→	Sports commitment	0.607	0.777	0.098	6.221 ***	0.382	0.478	0.105	3.633 ***
Physical self-efficacy	→	Exercise adherence	0.249	0.327	0.105	2.362	0.663	0.529	0.161	4.110 ***
Physical self-efficacy	→	Sports commitment	0.231	0.327	0.096	2.405	0.708	0.625	0.175	4.036 ***

*** *p* < 0.001; tested using structural equation modeling.

## Data Availability

The data supporting the findings of this study are available from the corresponding author upon request.
